# Functional Development of the Circadian Clock in the Zebrafish Pineal Gland

**DOI:** 10.1155/2014/235781

**Published:** 2014-04-16

**Authors:** Zohar Ben-Moshe, Nicholas S. Foulkes, Yoav Gothilf

**Affiliations:** ^1^Department of Neurobiology, George S. Wise Faculty of Life Sciences and Sagol School of Neuroscience, Tel-Aviv University, 69978 Tel-Aviv, Israel; ^2^Institute of Toxicology and Genetics, Karlsruhe Institute of Technology, 76344 Eggenstein-Leopoldshafen, Germany

## Abstract

The zebrafish constitutes a powerful model organism with unique advantages for investigating the vertebrate circadian timing system and its regulation by light. In particular, the remarkably early and rapid development of the zebrafish circadian system has facilitated exploring the factors that control the onset of circadian clock function during embryogenesis. Here, we review our understanding of the molecular basis underlying functional development of the central clock in the zebrafish pineal gland. Furthermore, we examine how the directly light-entrainable clocks in zebrafish cell lines have facilitated unravelling the general mechanisms underlying light-induced clock gene expression. Finally, we summarize how analysis of the light-induced transcriptome and miRNome of the zebrafish pineal gland has provided insight into the regulation of the circadian system by light, including the involvement of microRNAs in shaping the kinetics of light- and clock-regulated mRNA expression. The relative contributions of the pineal gland central clock and the distributed peripheral oscillators to the synchronization of circadian rhythms at the whole animal level are a crucial question that still remains to be elucidated in the zebrafish model.

## 1. Introduction

Many aspects of animal behaviour and physiology change significantly over the course of the day-night cycle. This phenomenon confers a selective advantage in relation to changing environmental factors such as food availability, predation risk, temperature, and light [1]. Accordingly, animals have evolved an intrinsic timing mechanism, the circadian clock, which drives day-night rhythms in physiology and behaviour. This clock is reset (“entrained”) on a daily basis by environmental signals, primarily light, to ensure synchronization of endogenous rhythms with the 24-hour solar day [2].

Circadian clock research has encompassed essentially all life forms, from the most primitive to the most advanced [3]. Nevertheless, most studies have been conducted in “traditional” genetic models such as the fruit fly and mouse [4, 5]. These studies have revealed the molecular components of the circadian clock, which function essentially in every cell and are coordinated by a master clock that resides in the brain [6]. Amongst vertebrates, the zebrafish represents a powerful model organism with unique advantages for exploring the mechanisms of the circadian clock and its entrainment by light [7]. In particular, as reviewed here, the zebrafish provides access to a circadian timing system that emerges remarkably early during development, a valuable feature for investigation of the functional development of the circadian clock.

## 2. The Pineal Gland and Rhythmic Melatonin Production

One major output of the vertebrate circadian clock is the rhythmic synthesis and secretion of the pineal gland hormone, melatonin, which constitutes an essential component of the circadian timing system. Being produced at night, melatonin provides a night-time signal and plays an endocrine role in the regulation of a variety of daily and annual physiological rhythms [8]. Classical examples come from studies in hamsters and sheep, in which the duration of melatonin secretion has been shown to control seasonal changes in reproduction and energy balance via basal hypothalamic sites [9].

The rate of melatonin production is determined by the enzymatic activity of arylalkylamine-*N*-acetyltransferase (AANAT). High melatonin levels at night reflect increased AANAT synthesis and activity, while the termination of melatonin production during the day reflects proteasomal degradation of this enzyme [10, 11]. In nearly all vertebrate species, AANAT activity and melatonin production in the pineal gland are controlled by the circadian clock and modulated by external photic signals. In mammals, the oscillations of AANAT activity and melatonin production are driven by the suprachiasmatic nucleus (SCN) of the hypothalamus [12], which functions as the master clock that coordinates the peripheral cellular oscillators [6]. Neurons of the SCN, which produce a circadian rhythm of firing rate, communicate time and photic information to the pineal gland indirectly through a multisynaptic neural pathway. At night, the SCN stimulates the release of norepinephrine in the pineal gland, generating increased pineal cAMP levels, leading to the phosphorylation of AANAT, which activates AANAT and protects it from proteasomal proteolysis [13, 14]. The suppressive effects of light are achieved by decreased cAMP levels, followed by a rise in dephosphorylated AANAT, leading to its inactivation and rapid proteolytic destruction [15]. In summary, pineal* aanat *has been established as an essential link between the vertebrate circadian clock and its important output signal—melatonin.

## 3. The Fish Pineal Gland Melatonin System

**Figure 1:**
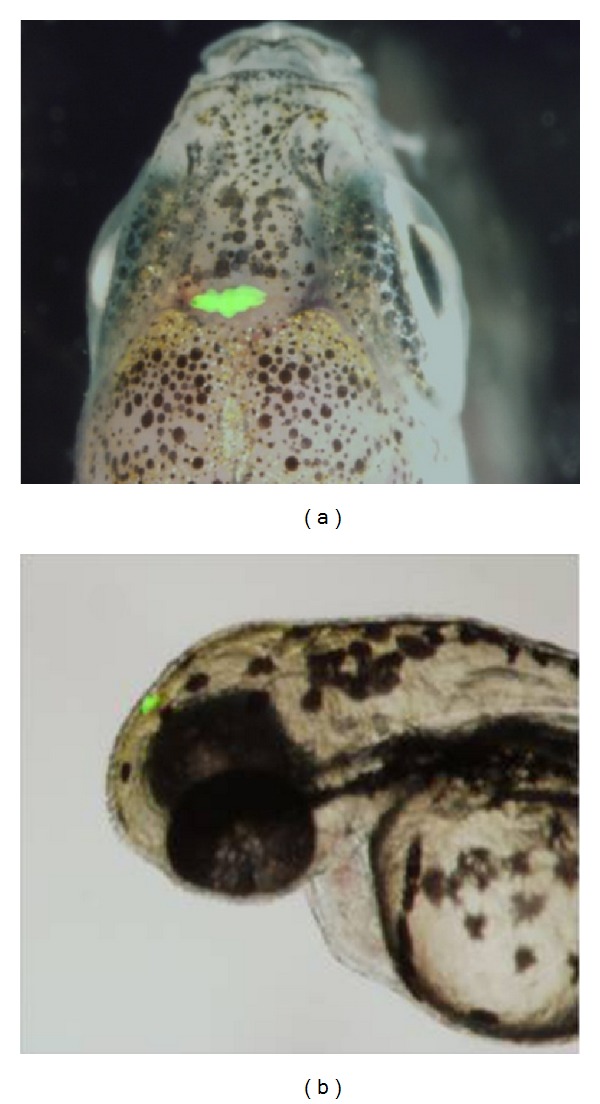
The zebrafish pineal gland. Transgenic zebrafish expressing enhanced green fluorescent protein (EGFP) in the pineal gland under the control of the pineal-specific* aanat2* promoter [21]. (a) The head region of an adult zebrafish, dorsal view, anterior to the top. (b) The head region of a 72 hpf zebrafish larva, lateral view, anterior to the left.

To date, a homologous structure to the mammalian SCN has not been identified in fish. However, as is the case in other nonmammalian vertebrates, the fish pineal gland (Figure 1) incorporates all the elements required for photic entrainment and circadian rhythm generation: it is photoreceptive and contains an intrinsic circadian oscillator that drives melatonin rhythms [16]. These basic properties are even maintained in culture, when the pineal gland is disconnected from any neuronal input [17]. In some nonmammalian vertebrates, the pineal gland is considered to serve as the master clock organ because its removal results in disruption of rhythmic behaviours such as locomotor activity [18, 19]. Thus, the pineal gland is thought to have evolved from a photoneuroendocrine structure that contains an independent clock, as seen in teleost fish, into an endocrine gland that is driven by SCN neuronal signals in mammals [20].

The roles of the pineal gland and of melatonin in fish have been traditionally investigated by pinealectomy and exogenous administration of melatonin, providing evidence for their role in seasonal reproduction and daily rhythms. However, given the incredible diversity among teleosts, it is not surprising that studies in different fish species have produced conflicting results, ranging from no effect of pinealectomy or melatonin administration to the loss of annual and daily physiological and behavioural rhythms, making it difficult to draw a general conclusion about the role of the pineal gland and melatonin in fish physiology [20, 22, 23]. Moreover, interpretation of the results is challenging. First, in addition to the melatonin-producing photoreceptor cells, the fish pineal gland contains projecting neurons [24–26] that innervate a variety of brain regions [27] and could therefore potentially transmit photic and/or circadian information. Therefore, pinealectomy eliminates both hormonal and neuronal signals. Second, melatonin is also produced in the retina, and although the role of retinal melatonin is considered to be restricted to paracrine effects, this inevitably implies that pinealectomy is an insufficient test of the general role of melatonin. Third, exogenous melatonin administration can be misleading because the effects of melatonin depend on its duration and circadian timing. Hence, further research is required to assess the role of the fish pineal gland and melatonin rhythms in coordinating circadian and annual rhythms of physiology and behaviour.

## 4. Early Development of the Zebrafish Circadian System

Among the advantages of the zebrafish model for experimental manipulation are its small size and ease of maintenance in large numbers, its short generation time and high fecundity, its external fertilization, and the rapid development of transparent embryos. Furthermore, the zebrafish model offers a plethora of molecular-genetic techniques and bioinformatics tools, including methods for transgenesis, mutagenesis, gene knockdown, and targeted genome modifications, together with advanced genomic annotation.

When it comes to circadian biology, another advantage of the zebrafish, especially for studying the role of melatonin in the regulation of circadian rhythms, is that, like humans, this species is diurnal. The role of the pineal gland and the effects of melatonin on different developmental, physiological, and behavioural processes have been studied in zebrafish by several research groups [28–32]. Among their findings, these studies have shown that exogenous melatonin administration leads to reduced locomotor activity and promotes a sleep-like state [33–35] and acts to schedule the timing of reproduction [28] and feeding [31].

Physiological and behavioural rhythms in zebrafish appear early in life. Circadian rhythms of nocturnal sleep-like behaviour [35] and diurnal locomotor activity [36–38], as well as circadian rhythms of respiration, posture and arousal threshold [35, 39], are established in zebrafish larvae at 4-5 days postfertilization (dpf). In addition, waves of cell cycles in the skin are apparent in zebrafish larvae by 4-5 dpf [40]. In* per3*-luc transgenic zebrafish, rhythmic luciferase activity in the whole body is evident at 5-6 dpf [41]. Importantly, the establishment of all of these behavioural, physiological, and molecular rhythms has been shown to require exposure of the larvae to light-dark cycles. This remarkably early and rapid development of the zebrafish circadian timing system is particularly intriguing and has been instrumental for the investigation of this system, for example, by enabling functional analyses of genes in intact developing fish.

A circadian rhythm that is established even earlier is that of* aanat2* expression in the pineal gland. The zebrafish, like other teleosts, possesses two* aanat* genes:* aanat1*, which is primarily expressed in the retina, and* aanat2*, which is predominantly expressed in the pineal gland [42, 43]. As in the case of other fish species [44], circadian rhythmicity of AANAT2 activity and melatonin production can be observed in cultured zebrafish pineal glands [45]. In addition, pineal* aanat2* transcription exhibits a robust circadian rhythm that is regulated by the core molecular oscillator, and its enzymatic activity and melatonin production are suppressed in response to light [43, 46–49]. The expression of* aanat2 *as well as other pineal gland markers first appears as early as 22 hours postfertilization (hpf), and the circadian rhythms of melatonin production and of* aanat2* transcription begin at 2 dpf, triggered by exposure to light [43, 50–52]. Importantly, this well-documented, robust clock-controlled gene expression and melatonin synthesis in the zebrafish embryonic pineal gland require exposure to a period of light, leading to the hypothesis that light exposure is mandatory for the development of overt clock-controlled rhythms in the pineal gland.

## 5. Light-Induced Onset of Circadian Rhythms in Zebrafish

What is the molecular mechanism underlying the light-induced onset of the pineal clock? In zebrafish embryos, light exposure induces the expression of* per2* mRNA predominantly in the pineal gland. Pineal gland* per2* mRNA levels increase rapidly following light onset, reaching a peak after 3 hours, while they remain undetectable under constant darkness [48]. Importantly, knockdown of* per2* abolishes* aanat2* mRNA rhythms in the pineal gland, indicating that light-induced* per2* expression is an important event in the developmental maturation of the pineal clock [48].

What are the photopigments that convey photic signals into the pineal gland oscillator? The teleost pineal gland is a classical photoreceptor organ that is evolutionarily and developmentally related to the retina [22] and expresses similar sets of genes, including opsins [42]. In the adult zebrafish,* exo-rhodopsin* is predominantly expressed in the pineal gland photoreceptors, along with several other extraretinal opsins [49, 53–55]. The expression of* exo-rhodopsin* in the zebrafish pineal gland is observed as early as 18 hpf [52] and it displays a daily rhythm, with higher mRNA levels during the night [54]. Furthermore,* exo-rhodopsin* has been shown to be required for high levels of* aanat2* transcription [54]. Therefore, light-induced* per2* expression in the pineal gland is most likely mediated by the early expressed pineal photopigment* exo-rhodopsin*.

Interestingly, light exposure also induces* per2* expression in nonpineal tissues, even at early developmental stages, prior to pineal gland or retina formation, indicating that light induces transcription in embryonic cells that are not considered classical photoreceptor cells [56, 57]. Moreover, exposure to light at these early developmental stages results in overt* aanat2* mRNA rhythms in the pineal gland at later stages, indicating that light-entrainment is preserved throughout proliferation and differentiation [57]. Indeed, it is now widely accepted that the molecular clocks within most zebrafish tissues and even cell lines are entrainable by direct exposure to light, and cell-based assays have been developed and used to study the mechanisms underlying light-induced gene expression [58–60].

The onset of rhythms in the zebrafish pineal gland is considered to represent the earliest essential light-entrainment event. This notion is supported by the observation of intermediate levels of* aanat2 *in the pineal gland of arrhythmic embryos that were not exposed to light during development [48]. Thus, in the absence of entraining cues, independent cellular oscillators in the pineal gland are out of phase, generating an overall intermediate level of* aanat2* expression. The synchronizing effect of light has also been demonstrated in a zebrafish cell line, in which a light pulse entrained the circadian oscillations of* per1b* promoter activity in individual cells and stabilized their 24-hour period, leading to a synchronized, overt rhythm of clock gene expression in the whole cell culture [58, 61]. This was further supported by the finding of asynchronous oscillations of the* per1b* transcript in individual cells of intact embryos raised in constant darkness [62]. Accordingly, in the developing circadian system, light input leads to the synchronization of preexisting cellular oscillators and not to their initial activation, resulting in the emergence of overt rhythms.

## 6. Mechanisms of Light-Induced Clock Gene Expression in Zebrafish

In order to explore the mechanisms underlying synchronization by light, the regulation of the light-induced zebrafish clock gene,* per2*, has been investigated. The regulation of the zebrafish* per2* promoter was first analyzed* in vivo*, leading to the identification of a minimal promoter fragment that is sufficient to drive* per2* expression and, importantly, regulation by light [63]. The existence of a photoentrainable clock system within zebrafish cells has greatly facilitated the unravelling of the regulatory mechanism underlying the light-induced* per2* expression. These* ex vivo* studies in zebrafish Pac-2 cells revealed a novel molecular mechanism that simultaneously drives clock- and light-regulated transcription [63]. This mechanism is mediated by closely spaced E-box and D-box regulatory elements that are located in proximity to the* per2* transcription start site [63]. The light-induced transcriptional activation was shown to be mediated by the D-box element and a D-box binding transcription factor,* tef-1 *[63]. Eleven additional zebrafish D-box-binding factors from the PAR and E4BP4 family have since been cloned and characterized. The expression of nine of these factors is enhanced in the pineal gland and regulated, to varying extents, by the clock and/or by light [64]. Moreover, it was demonstrated that the expression of some of these factors exhibits a somewhat similar clock- or light-driven regulation in zebrafish Pac-2 cells [65]. A systematic functional analysis of the* cry1a* promoter revealed that a single D-box directs light-induced expression of this clock gene and that PAR factors are able to transactivate expression from this D-box element [65]. The D-box-mediated pathway has also been implicated in the regulation of other light-induced genes [66, 67]. Hence, D-box enhancers appear to serve as key elements in light-driven signalling in both the pineal gland and cell lines, pointing towards a somewhat similar mechanism of light-entrainment in the central and peripheral clocks. Interestingly, this differs from the situation in the mammalian circadian timing system, where D-boxes appear to serve as regulatory elements of clock output pathways [68]. The D-box-mediated pathway is probably not the only mechanism underlying light-entrainment of the circadian oscillator in fish. Might a genome-wide approach lead to the identification of parallel mechanisms?

## 7. Insights from the Light-Induced Transcriptome of the Zebrafish Pineal Gland

Similar to the studies of many other biological processes, circadian clock research has greatly benefited from the availability of technologies for large-scale analysis of transcriptomes, including recent advances in high-throughput RNA sequencing (RNA-seq). These technologies have also been employed for studying the mechanisms underlying light-induced gene expression. In zebrafish, DNA microarray studies of the light-induced transcriptome of embryos [66], larvae, heart, and cell cultures [67] have identified numerous genes belonging to various cellular processes, including transcriptional control and DNA repair, which are directly light-regulated. These studies have expanded the knowledge of light-regulated gene expression in peripheral clock-containing tissues.

**Figure 2:**
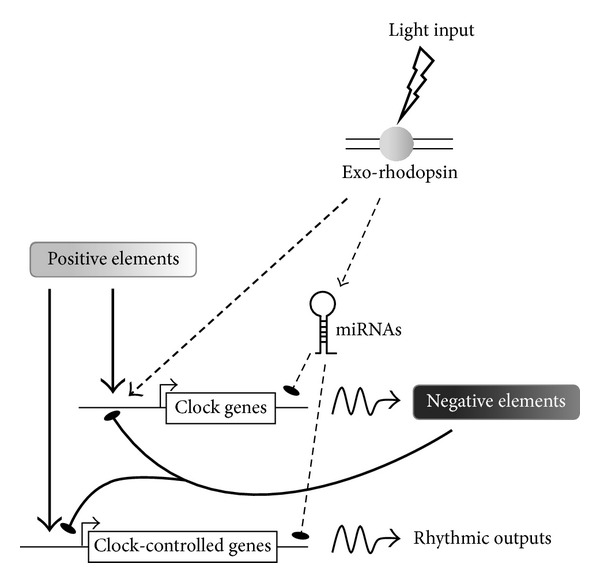
Model of the regulation of the molecular clockwork in the zebrafish pineal gland by light. Light input is perceived by exo-rhodopsin at the cell membrane and relayed to the nucleus by signal transduction pathways. The light signal upregulates the expression of negative elements in the clockwork circuitry. In addition, light-induced miRNAs contribute to the generation of transient expression profiles of clock and clock-controlled target genes. Arrows indicate activation; lines with flat end indicate inhibition.

With the aim of further exploring the mechanisms by which the central circadian clock is entrained by light, we employed both RNA-seq and microarray technologies to characterize the light-induced coding transcriptome of the zebrafish pineal gland [69], resulting in the identification of multiple light-induced mRNAs. An interesting outcome of this approach was the identification of 14-core clock and clock accessory loop genes as light-induced genes in the pineal gland, including* per2* and* cry1a*, most of which are members of the negative limbs of the molecular oscillator (Figure 2). The finding that a considerable portion of the molecular clock is regulated by light in the central clock structure points to a more complex regulatory mechanism underlying light-entrainment of the circadian clock than previously appreciated. This complexity has been further demonstrated by overexpression analyses of four of these genes, encoding the transcription factors* dec1*,* reverb*β*1*,* e4bp4-5,* and* e4bp4-6*, in zebrafish Pac-2 cells. These analyses revealed different effects of the factors on clock and light-regulated promoter activation, demonstrating various mechanisms by which light-induced transcription factors modulate clock gene expression and thereby transmit photic information to the core clock. Moreover, we have shown that* dec1* is important for the light-induced onset of rhythmic locomotor activity in zebrafish larvae. This was achieved by the knockdown of* dec1*, which resulted in the disruption of circadian locomotor activity patterns triggered by a single light pulse, resembling the effect generated by* per2* knockdown. A previous study in* dec1*-deficient mice provided evidence for its role in the resetting of the circadian clock [70]; thus, our data indicate an equivalent role for* dec1* in the process of light-entrainment of the zebrafish clock.

Another intriguing finding was that cellular metabolic pathways are induced by light. Of particular interest is the finding that the expression of* hypoxia-inducible factor 1*α** (*hif1*α**) and its target* pfkfb4l* are both induced by light, which points to the possibility that the hypoxic pathway is involved in circadian clock entrainment, a hypothesis that requires further investigation.

A common feature of light-induced genes, as well as rhythmic genes, is their transient increase in expression, suggesting the contribution of mechanisms that control mRNA stability, such as regulation by microRNA (miRNA). To search for candidate miRNAs that might play a role in light-entrainment and circadian regulation, we exploited miRNA sequencing (miR-seq) to profile the repertoire of light-induced and abundant miRNAs in the zebrafish pineal gland [69]. This analysis implicated the miR-183/96/182 cluster, the expression of which is both considerably enriched in the pineal gland and upregulated by light, in the regulation of transiently expressed mRNAs. This miRNA cluster has previously been shown to display a daily variation of expression in the mouse retina. Furthermore, it has been suggested to play a role in circadian rhythm regulation via its targeting of* adcy6*, a clock-controlled gene that modulates melatonin synthesis [71]. In a later study, the miR-183/96/182 cluster was found to be regulated by light in the mouse retina and to target the voltage-dependent glutamate transporter* slc1a1* [72]. The miR-183/96/182 cluster has also been shown to be abundantly expressed in the rat retina and pineal gland, in which it exhibits daily dynamics of expression [73]. We have demonstrated that miR-183 downregulates the light-induced* e4bp4-6 *and clock-controlled* aanat2* mRNAs via target sites in their 3′UTR regions and, importantly,* in vivo* knockdown analysis indicates that miR-183 contributes to the generation of* aanat2* rhythmic mRNA levels in the pineal gland. Together, these findings imply a conserved function for the miR-183/96/182 cluster in vertebrates and support its involvement in regulating the kinetics of both clock- and light-regulated gene expression (Figure 2). The essential contribution of miRNAs to shaping the transient expression profiles of light- and clock-regulated genes, and their general importance in pineal function, such as fine-tuning the kinetics of rhythmic melatonin production, warrants further investigation.

## 8. Concluding Remarks

The remarkably early development of the zebrafish circadian clock in the pineal gland has provided a unique opportunity for a thorough investigation of this timing system and has led to the discovery of mechanisms that underlie its maturation. One important conclusion has been that the light-induced onset of the circadian clock is actually a specific case of entrainment of asynchronous cellular oscillators. The analysis of the regulatory mechanisms underlying light-induced clock gene expression in photoentrainable zebrafish cell lines has served as an additional important step towards unravelling the process of circadian clock light-entrainment. Another key step forward in our understanding of these light-regulated mechanisms has been achieved by genome-wide analyses of the light-induced transcriptome in zebrafish embryos, larvae, heart, and cell lines and within the pineal gland. Analysis of the pineal gland light-induced transcriptome indicated that the regulation of the circadian system by light is rather complex, involving multiple factors and pathways. Analysis of the pineal-enhanced and light-induced miRNome has revealed the contribution of miRNAs to light- and clock-regulated expression and to pineal function. There are clear similarities between the central and peripheral clocks in terms of their basic mechanisms and regulation by light. The relative contributions of the pineal gland clock and the distributed peripheral oscillators to synchronizing the physiology and behaviour of the intact animal with the day-night cycle present an important question that still remains to be addressed in the zebrafish model.

## References

[1] Pittendrigh CS (1993). Temporal organization: reflections of a Darwinian clock-watcher. *Annual Review of Physiology*.

[2] Doyle S, Menaker M (2007). Circadian photoreception in vertebrates. *Cold Spring Harbor Symposia on Quantitative Biology*.

[3] Dunlap JC (1999). Molecular bases for circadian clocks. *Cell*.

[4] Peschel N, Helfrich-Förster C (2011). Setting the clock—by nature: circadian rhythm in the fruitfly *Drosophila melanogaster*. *FEBS Letters*.

[5] Ripperger JA, Jud C, Albrecht U (2011). The daily rhythm of mice. *FEBS Letters*.

[6] Dibner C, Schibler U, Albrecht U (2010). The mammalian circadian timing system: organization and coordination of central and peripheral clocks. *Annual Review of Physiology*.

[7] Vatine G, Vallone D, Gothilf Y, Foulkes NS (2011). It’s time to swim! Zebrafish and the circadian clock. *FEBS Letters*.

[8] Arendt J (1998). Melatonin and the pineal gland: influence on mammalian seasonal and circadian physiology. *Reviews of Reproduction*.

[9] Morgan PJ, Hazlerigg DG (2008). Photoperiodic signalling through the melatonin receptor turns full circle. *Journal of Neuroendocrinology*.

[10] Falcón J, Galarneau KM, Weller JL (2001). Regulation of arylalkylamine *N*-acetyltransferase-2 (AANAT2, EC 2.3.1.87) in the fish pineal organ: evidence for a role of proteasomal proteolysis. *Endocrinology*.

[11] Gastel JA, Roseboom PH, Rinaldi PA, Weller JL, Klein DC (1998). Melatonin production: proteasomal proteolysis in serotonin *N*-acetyltransferase regulation. *Science*.

[12] Klein DC, Coon SL, Roseboom PH (1997). The melatonin rhythm-generating enzyme: molecular regulation of serotonin *N*-acetyltransferase in the pineal gland. *Recent Progress in Hormone Research*.

[13] Ganguly S, Coon SL, Klein DC (2002). Control of melatonin synthesis in the mammalian pineal gland: the critical role of serotonin acetylation. *Cell and Tissue Research*.

[14] Schomerus C, Korf H-W, Laedtke E, Weller JL, Klein DC (2000). Selective adrenergic/cyclic AMP-dependent switch-off of proteasomal proteolysis alone switches on neural signal transduction: an example from the pineal gland. *Journal of Neurochemistry*.

[15] Klein DC, Ganguly S, Coon S (2002). 14-3-3 proteins and photoneuroendocrine transduction: role in controlling the daily rhythm in melatonin. *Biochemical Society Transactions*.

[16] Korf HW, Schomerus C, Stehle JH (1998). The pineal organ, its hormone melatonin, and the photoneuroendocrine system. *Advances in Anatomy, Embryology, and Cell Biology*.

[17] Falcon J, Marmillon JB, Claustrat B, Collin J-P (1989). Regulation of melatonin secretion in a photoreceptive pineal organ: an in vitro study in the pike. *Journal of Neuroscience*.

[18] Gwinner E, Hau M, Heigl S (1997). Melatonin: generation and modulation of avian circadian rhythms. *Brain Research Bulletin*.

[19] Underwood H (1983). Circadian pacemakers in lizards: phase-response curves and effects of pinealectomy. *American Journal of Physiology*.

[20] Falcón J, Besseau L, Fuentès M, Sauzet S, Magnanou E, Boeuf G (2009). Structural and functional evolution of the pineal melatonin system in vertebrates. *Annals of the New York Academy of Sciences*.

[21] Gothilf Y, Toyama R, Coon SL, Du S-J, Dawid IB, Klein DC (2002). Pineal-specific expression of green fluorescent protein under the control of the serotonin-*N*-acetyltransferase gene regulatory regions in transgenic zebrafish. *Developmental Dynamics*.

[22] Ekström P, Meissl H (1997). The pineal organ of teleost fishes. *Reviews in Fish Biology and Fisheries*.

[23] Falcón J, Migaud H, Muñoz-Cueto JA, Carrillo M (2010). Current knowledge on the melatonin system in teleost fish. *General and Comparative Endocrinology*.

[24] Masai I, Heisenberg C-P, Barth KA, Macdonald R, Adamek S, Wilson SW (1997). Floating head and masterblind regulate neuronal patterning in the roof of the forebrain. *Neuron*.

[25] Wilson SW, Easter SS (1991). A pioneering growth cone in the embryonic zebrafish brain. *Proceedings of the National Academy of Sciences of the United States of America*.

[26] Wilson SW, Ross LS, Parrett T, Easter SS (1990). The development of a simple scaffold of axon tracts in the brain of the embryonic zebrafish, *Brachydanio rerio*. *Development*.

[27] Yáñez J, Busch J, Anadón R, Meissl H (2009). Pineal projections in the zebrafish (*Danio rerio*): overlap with retinal and cerebellar projections. *Neuroscience*.

[28] Carnevali O, Gioacchini G, Maradonna F, Olivotto I, Migliarini B (2011). Melatonin induces follicle maturation in *Danio rerio*. *PLoS ONE*.

[29] Danilova N, Krupnik VE, Sugden D, Zhdanova IV (2004). Melatonin stimulates cell proliferation in zebrafish embryo and accelerates its development. *The FASEB Journal*.

[30] de Borsetti NH, Dean BJ, Bain EJ, Clanton JA, Taylor RW, Gamse JT (2011). Light and melatonin schedule neuronal differentiation in the habenular nuclei. *Developmental Biology*.

[31] Piccinetti CC, Migliarini B, Olivotto I, Coletti G, Amici A, Carnevali O (2010). Appetite regulation: the central role of melatonin in *Danio rerio*. *Hormones and Behavior*.

[32] Rawashdeh O, de Borsetti NH, Roman G, Cahill GM (2007). Melatonin suppresses nighttime memory formation in zebrafish. *Science*.

[33] Zhdanova IV (2006). Sleep in zebrafish. *Zebrafish*.

[34] Zhdanova IV (2011). Sleep and its regulation in zebrafish. *Reviews in the Neurosciences*.

[35] Zhdanova IV, Wang SY, Leclair OU, Danilova NP (2001). Melatonin promotes sleep-like state in zebrafish. *Brain Research*.

[36] Cahill GM (2007). Automated video image analysis of larval zebrafish locomotor rhythms. *Methods in Molecular Biology*.

[37] Hirayama J, Kaneko M, Cardone L, Cahill G, Sassone-Corsi P (2005). Analysis of circadian rhythms in zebrafish. *Methods in Enzymology*.

[38] Hurd MW, Cahill GM (2002). Entraining signals initiate behavioral circadian rhythmicity in larval zebrafish. *Journal of Biological Rhythms*.

[39] Prober DA, Rihel J, Onah AA, Sung R-J, Schier AF (2006). Hypocretin/orexin overexpression induces an insomnia-like phenotype in zebrafish. *Journal of Neuroscience*.

[40] Dekens MPS, Santoriello C, Vallone D, Grassi G, Whitmore D, Foulkes NS (2003). Light regulates the cell cycle in zebrafish. *Current Biology*.

[41] Kaneko M, Cahill GM (2005). Light-dependent development of circadian gene expression in transgenic zebrafish. *PLoS Biology*.

[42] Falcón J, Gothilf Y, Coon SL, Boeuf G, Klein DC (2003). Genetic, temporal and developmental differences between melatonin rhythm generating systems in the teleost fish pineal organ and retina. *Journal of Neuroendocrinology*.

[43] Gothilf Y, Coon SL, Toyama R, Chitnis A, Namboodiri MAA, Klein DC (1999). Zebrafish serotonin *N*-acetyltransferase-2: marker for development of pineal photoreceptors and circadian clock function. *Endocrinology*.

[44] Falcón J (1999). Cellular circadian clocks in the pineal. *Progress in Neurobiology*.

[45] Cahill GM (1996). Circadian regulation of melatonin production in cultured zebrafish pineal and retina. *Brain Research*.

[46] Appelbaum L, Anzulovich A, Baler R, Gothilf Y (2005). Homeobox-clock protein interaction in zebrafish: a shared mechanism for pineal-specific and circadian gene expression. *The Journal of Biological Chemistry*.

[47] Bégay V, Falcón J, Cahill GM, Klein DC, Coon SL (1998). Transcripts encoding two melatonin synthesis enzymes in the teleost pineal organ: circadian regulation in pike and zebrafish, but not in trout. *Endocrinology*.

[48] Ziv L, Levkovitz S, Toyama R, Falcon J, Gothilf Y (2005). Functional development of the zebrafish pineal gland: light-induced expression of period2 is required for onset of the circadian clock. *Journal of Neuroendocrinology*.

[49] Ziv L, Tovin A, Strasser D, Gothilf Y (2007). Spectral sensitivity of melatonin suppression in the zebrafish pineal gland. *Experimental Eye Research*.

[50] Gamse JT, Shen Y-C, Thisse C (2002). Otx5 regulates genes that show circadian expression in the zebrafish pineal complex. *Nature Genetics*.

[51] Kazimi N, Cahill GM (1999). Development of a circadian melatonin rhythm in embryonic zebrafish. *Developmental Brain Research*.

[52] Vuilleumier R, Besseau L, Boeuf G (2006). Starting the zebrafish pineal circadian clock with a single photic transition. *Endocrinology*.

[53] Mano H, Kojima D, Fukada Y (1999). Exo-rhodopsin: a novel rhodopsin expressed in the zebrafish pineal gland. *Molecular Brain Research*.

[54] Pierce LX, Noche RR, Ponomareva O, Chang C, Liang JO (2008). Novel functions for period 3 and Exo-rhodopsin in rhythmic transcription and melatonin biosynthesis within the zebrafish pineal organ. *Brain Research*.

[55] Toyama R, Chen X, Jhawar N (2009). Transcriptome analysis of the zebrafish pineal gland. *Developmental Dynamics*.

[56] Tamai TK, Vardhanabhuti V, Foulkes NS, Whitmore D (2004). Early embryonic light detection improves survival. *Current Biology*.

[57] Ziv L, Gothilf Y (2006). Circadian time-keeping during early stages of development. *Proceedings of the National Academy of Sciences of the United States of America*.

[58] Carr A-JF, Tamai TK, Young LC, Ferrer V, Dekens MP, Whitmore D (2006). Light reaches the very heart of the zebrafish clock. *Chronobiology International*.

[59] Vallone D, Lahiri K, Dickmeis T, Foulkes NS (2005). Zebrafish cell clocks feel the heat and see the light!. *Zebrafish*.

[60] Vallone D, Santoriello C, Gondi SB, Foulkes NS (2007). Basic protocols for zebrafish cell lines: maintenance and transfection. *Methods in Molecular Biology*.

[61] Carr A-JF, Whitmore D (2005). Imaging of single light-responsive clock cells reveals fluctuating free-running periods. *Nature Cell Biology*.

[62] Dekens MPS, Whitmore D (2008). Autonomous onset of the circadian clock in the zebrafish embryo. *The EMBO Journal*.

[63] Vatine G, Vallone D, Appelbaum L (2009). Light directs zebrafish period2 expression via conserved D and E boxes. *PLoS Biology*.

[64] Ben-Moshe Z, Vatine G, Alon S (2010). Multiple PAR and E4BP4 bZIP transcription factors in zebrafish: diverse spatial and temporal expression patterns. *Chronobiology International*.

[65] Mracek P, Santoriello C, Idda ML (2012). Regulation of *per* and *cry* genes reveals a central role for the D-box enhancer in light-dependent gene expression. *PLoS ONE*.

[66] Gavriouchkina D, Fischer S, Ivacevic T, Stolte J, Benes V, Dekens MPS (2010). Thyrotroph embryonic factor regulates light-induced transcription of repair genes in zebrafish embryonic cells. *PLoS ONE*.

[67] Weger BD, Sahinbas M, Otto GW (2011). The light responsive transcriptome of the zebrafish: function and regulation. *PLoS ONE*.

[68] Ripperger JA, Shearman LP, Reppert SM, Schibler U (2000). CLOCK, an essential pacemaker component, controls expression of the circadian transcription factor DBP. *Genes & Development*.

[69] Ben-Moshe Z, Alon S, Mracek P (2014). The light-induced transcriptome of the zebrafish pineal gland reveals complex regulation of the circadian clockwork by light. *Nucleic Acids Research*.

[70] Rossner MJ, Oster H, Wichert SP (2008). Disturbed clockwork resetting in sharp-1 and sharp-2 single and double mutant mice. *PLoS ONE*.

[71] Xu S, Witmer PD, Lumayag S, Kovacs B, Valle D (2007). MicroRNA (miRNA) transcriptome of mouse retina and identification of a sensory organ-specific miRNA cluster. *The Journal of Biological Chemistry*.

[72] Krol J, Busskamp V, Markiewicz I (2010). Characterizing light-regulated retinal microRNAs reveals rapid turnover as a common property of neuronal microRNAs. *Cell*.

[73] Clokie SJ, Lau P, Kim HH, Coon SL, Klein DC (2012). MicroRNAs in the pineal gland: miR-483 regulates melatonin synthesis by targeting arylalkylamine *N*-acetyltransferase. *The Journal of Biological Chemistry*.

